# Health utility value of overactive bladder in Japanese older adults

**DOI:** 10.1002/bco2.471

**Published:** 2024-11-24

**Authors:** Takashi Yoshioka, Kenji Omae, Satoshi Funada, Tetsuji Minami, Rei Goto

**Affiliations:** ^1^ Health Technology Assessment Unit, Department of Preventive Medicine and Public Health Keio University School of Medicine Tokyo Japan; ^2^ Institute of Clinical Epidemiology Showa University Tokyo Japan; ^3^ Department of Innovative Research and Education for Clinicians and Trainees (DiRECT) Fukushima Medical University Hospital Fukushima Japan; ^4^ Department of Health Promotion and Human Behavior, Graduate School of Medicine/School of Public Health Kyoto University Kyoto Japan; ^5^ Division of Quality Assurance Programs Institute for Cancer Control, National Cancer Center Tokyo Japan; ^6^ Graduate School of Health Management Keio University Kanagawa Japan; ^7^ Graduate School of Business Administration Keio University Kanagawa Japan

**Keywords:** health economic evaluation, health utility, health‐related quality of life, lower urinary tract symptoms, overactive bladder

## Abstract

**Objectives:**

To determine the health utility values (HUVs) of overactive bladder (OAB), defined as urinary urgency, usually accompanied by urinary daytime or nocturnal frequency, with or without urinary incontinence, among adults aged ≥65 years and to assess the HUV decrements (disutilities) of OAB according to its severity.

**Methods:**

This cross‐sectional Internet‐based study was conducted between 2 and 9 November 2023, with quota sampling with equal probability for each sex and age group (age 65–74 years and ≥75 years). OAB was defined as an urgency score of ≥2 points and a total score of ≥3 points based on the Overactive Bladder Symptom Score. OAB severity was categorized as mild (total score, ≤5 points) or moderate‐to‐severe (total score, 6–15 points). HUVs were measured using the EuroQol five‐dimension five‐level value set for the Japanese population. Multivariable linear regression models were fitted to estimate the covariate‐adjusted disutilities of OAB. We selected eight covariates (age, sex, body mass index, education, income, smoking, alcohol use, and comorbidities) as potential confounders based on previous studies. The sample size was determined based on previous studies without statistical power calculations.

**Results:**

Among the 998 participants (51.9% male; mean age, 73.2 years), 158 (15.9%) had OAB, of whom 87 (8.8%) had moderate‐to‐severe OAB. The mean HUVs for participants with mild and moderate‐to‐severe OAB were 0.874 and 0.840, respectively, which were lower compared with the HUV for those without OAB (0.913). After adjusting for relevant covariates, disutilities (95% confidence intervals [CIs] and *p* values) for mild and moderate‐to‐severe OAB were −0.0334 (−0.0602 to −0.0066, *p* = 0.014) and −0.0591 (−0.0844 to −0.0339, *p* < 0.001), respectively.

**Conclusions:**

Consistent with previous HUV studies on OAB, our results demonstrated that the prevalence of OAB was associated with substantially lower HUV. The results demonstrate that increased OAB severity is associated with greater disutility.

## 1 INTRODUCTION

Overactive bladder (OAB), characterized by urinary urgency with or without urgency urinary incontinence, is associated with a substantial disease burden and significant economic impact.^1^ The extensive disease burden of OAB includes a decrease in health‐related quality of life (HRQoL), mood disorders, and various comorbid or consequential conditions (e.g. constipation, faecal incontinence, and irritable bowel syndrome).^2^ The prevalence of OAB increases with age.^3^ Furthermore, evidence has demonstrated a longitudinal association between OAB or its treatment with anticholinergics and an increased risk of falls, which are the most critical outcomes in older adults.^4^, ^5^ Various comorbidities associated with OAB, as well as falls, contribute to patients with OAB having higher healthcare resource utilization than those without the condition, which contributes to a substantial economic burden.^6^ The high global prevalence of OAB, estimated at 15.5% between 2007 and 2020, and its high healthcare cost of $82.6 billion by 2020 in the United States^1^, ^7^ have drawn significant attention from both policymakers and clinicians worldwide.

OAB is managed in various healthcare settings, including primary care, urology, and urogynecology.^8^ Treatment options for OAB include behavioural therapy and pharmacotherapy, such as antimuscarinic and β‐adrenergic agents.^8^ Surgical treatments are available for refractory cases, including intradetrusor injection of onabotulinum toxin A, sacral neuromodulation, and percutaneous tibial nerve stimulation.^8^ Although these therapeutic options have the potential to relieve patients' symptoms and improve their HRQoL, they also raise concerns about the increasing economic burden on healthcare systems owing to higher treatment costs. Consequently, the importance of effectively allocating finite healthcare resources for the management of OAB is becoming increasingly recognized. In fact, the National Institute for Health and Care Excellence (NICE), an independent organization providing evidence‐based guidance to inform healthcare decisions in the United Kingdom (UK), is currently conducting a health economic evaluation (HEE) of vibegron,^9^ a novel β3‐adrenergic agonist with fewer antimuscarinic side effects compared with traditional OAB medications.^10^ Considering the example of the NICE in the UK, new medical technologies for OAB, such as vibegron, may also be subject to HEEs in other countries, such as Japan.

To make HEEs useful for policy decision‐making, it is essential to assess their clinical effectiveness (e.g. quality‐adjusted life years [QALYs]) using generic measures that can be collected for any disease. QALYs are calculated based on health utility values (HUVs) measured using generic preference‐based measures (PBMs) rather than disease‐specific ones.^11^ Importantly, according to the NICE technology appraisal guidance, HUVs should be calculated using the EuroQoL 5‐dimensions (EQ‐5D), with data collected directly from patients in the country where the evaluation is being conducted (in the case of NICE, the UK).^12^ Previous studies have evaluated the association between OAB symptoms and HUVs using EQ‐5D scores in the United States, United Kingdom, Spain, and South Korea.^13^, ^14^, ^15^ However, these studies have utilized the older version of the EQ‐5D, the three‐level EQ‐5D version (EQ‐5D‐3L), which raises concerns about the potential overestimation of positive effects and underestimation of negative effects.^16^ The newer version of the EQ‐5D, the five‐level EQ‐5D version (EQ‐5D‐5L), was developed to overcome the ceiling effects and low sensitivity inherent in EQ‐5D‐3L.^17^ Ideally, the estimation of HUVs based on EQ‐5D‐5L is essential. However, few studies have used this instrument.

To address the unmet needs of global policies regarding HEEs of OAB, this study aimed to describe the HUVs of patients with OAB and assess the impacts of OAB on HUVs using EQ‐5D‐5L among older adults, the most vulnerable and policy‐relevant subgroup, using an Internet‐based survey.

## 2 PARTICIPANTS AND METHODS

### 2.1 Study design and participants

This cross‐sectional study was conducted as part of a research project aimed at developing an EQ‐5D‐5L value set for the Japanese general older adults.^17^ Referring to the 2024 population statistics,^18^ the general Japanese population aged ≥65 has more people aged ≥75 compared to those aged 65–74 (65–74 years, 15.49 million vs. ≥75 years, 20.76 million), and the population aged 75 and over has a higher proportion of women (65–74 years, female ratio 52.1%; 75 years and over, female ratio 60.0%). The purpose of this study is to estimate HUVs for older adults, requiring sufficient sample sizes for both sex and age groups (65–74 years, ≥75 years). Therefore, we used a sampling method that ensured a 1:1 ratio for both sex and age groups (age 65–74 and ≥75 years). The target sample size was 1000 participants. The sampling strategy and sample size were determined based on a previous study that developed a value set to convert EQ‐5D‐5L scores into HUVs.^19^ That study included 1026 participants with a 1:1 male‐to‐female ratio, sampling approximately 200 individuals from each 10‐year age group ranging from the 20s to the 60s. The sample size of the referenced study was not based on statistical power calculations.^19^, ^20^ We conducted online recruitment to access a wide age range of adults aged ≥65 years. All participants were recruited from a survey panel provided by a Japanese Internet research agency (Intage, Inc., https://www.intage.co.jp/english/) with approximately 2.6 million panellists and their demographic information. The survey was conducted between 2 and 9 November 2023. We designed the survey to prevent participants from proceeding to the next question without providing a valid response, thereby eliminating missing data. The survey questionnaire and response options are presented in Method S1.

### 2.2 Survey details

The survey collected demographic data (age, sex, height, and weight), which was anonymized prior to researcher access by the survey company. The survey instrument was developed by Intage Inc. The sampling methodology employed age‐ and sex‐based stratification, constituting a closed survey design. Data collection was conducted exclusively online among Intage Inc.'s panel registrants, who were authenticated through login credentials. Survey invitations were distributed via email and automatically recorded in Intage's system database. Participation was voluntary; potential participants received a study explanation at the outset and proceeded only after providing informed consent. The questionnaire maintained a fixed sequence without randomization. Partial adaptive questioning was implemented, whereby subsequent questions were suppressed following ‘prefer not to answer’ responses. The survey consisted of 27 questions, presented individually per page. No validation checks for response consistency or completeness were implemented. While navigation buttons (‘next’ and ‘back’) were available for the EQ‐5D‐5L and EQ VAS sections, the ‘back’ function was disabled for other questions. Unique respondent identification was achieved through cookies and IP address tracking. Of 14 849 individuals contacted, 1099 completed the survey, yielding a recruitment rate of 7.4%. The number of individuals who initially agreed to participate was not documented, precluding calculation of the response rate. Data regarding early termination and response interruptions were not available. While response time‐based exclusions were implemented, the specific time threshold was not disclosed.

### 2.3 Inclusion and exclusion criteria

All respondents were included in the analysis. However, participants who responded ‘prefer not to answer’ to at least one of the questionnaires except for income items were excluded from this study.

### 2.4 Exposures (overactive bladder)

The variable of interest in this study was the presence of OAB. OAB was assessed using the Overactive Bladder Symptom Score (OABSS), a validated questionnaire developed in Japanese.^21^ The OABSS is a self‐reported questionnaire consisting of four domains (daytime frequency, 0–2 points; nighttime frequency, 0–3 points; urgency, 0–5 points; urgency incontinence, 0–5 points), with a total score of 15 points^21^ A higher score indicates more severe OAB symptoms. Based on previous studies and decision modelling on HEEs, OAB was defined as an urgency score of ≥2 points and a total score of ≥3 points, and the severity of OAB was categorized as mild (OAB with a total score of ≤5 points) or moderate‐to‐severe (OAB with a total score of 6–15 points).^15^

### 2.5 Outcomes (health utility values)

Our primary outcome of interest was HUVs measured using the EQ‐5D‐5L instrument. HUVs are an indicator of an individual's current health state, where 0 represents death or the worst imaginable health state and 1 represents perfect health, with higher scores indicating better health status.^11^ In HEEs, HUVs are calculated using PBMs developed with choice methods (e.g. standard gambling or time trade‐off) as reference standards.^11^, ^12^ The EQ‐5D‐5L is a PBM developed by the EuroQoL Group that is used to measure HUVs based on time trade‐off methods.^11^, ^12^ The EQ‐5D‐5L comprises five dimensions, each with five levels. The dimensions are mobility, self‐care, usual activities, pain/discomfort, and anxiety/depression. The levels are no problems, slight problems, moderate problems, severe problems, and extreme problems. As the impact of each dimension and level on HUV varies by country, EQ‐5D‐5L scores are converted to HUVs based on country‐specific value sets.^22^ Importantly, the EQ‐5D‐5L has been translated and validated in Japanese, demonstrating substantial reliability and validity for calculating HUVs in the general Japanese population.^19^

In this study, we used the EuroQoL Visual Analogue Scale (EQ VAS) score as another measure of health utility. The EQ VAS is a scale developed by the EuroQoL Group that measures the current health state using a VAS on a vertically oriented ruler with markings from 0 to 100.^19^ On the EQ VAS, 0 represents death or the worst imaginable health state, and 100 indicates perfect health. The EQ VAS has often been used in conjunction with the EQ‐5D in similar studies,^15^ and consistent results have been reported. Therefore, we used the EQ VAS score as an outcome measure for sensitivity analysis in this study.

This study used the online formats of the EQ‐5D‐5L and EQ VAS. The research protocol was pre‐registered on the EuroQoL website,^17^ and permission was obtained to use these instruments. The online versions were administered in strict accordance with the manufacturers' instructions. The EuroQoL registration number for this study is 59 331.

### 2.6 Covariates

The covariates of interest in this study were demographic, socioeconomic, and health‐related variables, all of which are potential confounding factors in estimating the association between OAB and HUV. These variables were selected from previous studies on OAB that focused on the factors that could potentially influence HUVs. The demographic factors included age (continuous), sex (male or female), and body mass index BMI (continuous). Socioeconomic status included educational attainment (high school education or less and college education or more) and income.^7^ As a proxy for income, this study used equivalent household income, derived by adjusting total household income by the square root of household size (then grouped by median, with ranges provided: lower half, JPY 0–2.8 million; upper half, JPY ≥2.9 million), along with a declined to answer category. Health‐related factors included smoking status (never/past/current), alcohol consumption (never/past/current), and self‐reported coexisting physical and psychiatric comorbidities. Comorbidity data were used to record the presence or absence of the following diseases: hypertension, dyslipidaemia, diabetes, low back pain, ischaemic heart disease, stroke, chronic kidney disease, stress urinary incontinence, depression, and other psychiatric disorders.^6^, ^23^, ^24^, ^25^, ^26^

### 2.7 Statistical analyses

This study used three analytical approaches. First, a primary analysis was performed targeting all respondents. Second, subgroup analyses were performed, stratified based on sex (male/female) and age (65–74 years old/≥75 years), assuming that the impacts of OAB on HUVs may vary according to these factors. Third, an exploratory analysis was performed to evaluate the impact of each of the four domains constituting the OAB (daytime frequency, nighttime frequency, urgency, and urgency incontinence) on HUVs. In all analyses, we initially described the baseline characteristics, including the prevalence of OAB and HUVs. In the primary analysis, we divided the participants into two groups (those without OAB and those with OAB), described the baseline characteristics, and compared these two groups. For comparing each characteristic variable, we used Wilcoxon's rank sum test for continuous variables and Fisher's exact test for categorical variables. Subsequently, we described the mean (standard deviation [SD]) and median (interquartile range [IQR]) of HUVs based on summary statistics for each group for all analyses. In addition to describing summary statistics, we conducted univariate comparisons using a linear regression model, with HUV as the continuous outcome variable and the presence of OAB (or the presence of mild‐ or moderate‐to‐severe OAB) as the explanatory variable. Finally, we estimated the adjusted HUV decrements (disutilities) using a multivariable linear regression analysis after adjusting for covariates. In the multivariable linear regression analysis with ordinal categories (non‐OAB/mild OAB/moderate‐to‐severe OAB), a linear trend test was conducted to assess linearity. To confirm the consistency of the results, a sensitivity analysis was performed in the primary analysis to fit a similar linear regression model using the EQ VAS score as the outcome measure.

For all regression models, β coefficients, 95% confidence intervals (CIs), and *p* values were calculated. Statistical significance was set at *p* < 0.05. All analyses were performed using STATA version 18.0 (Stata Corp, College Station, TX, USA).

### 2.8 Ethical considerations

All respondents completed online questionnaires after providing digital informed consent indicating their willingness to participate in the survey. Participants received monetary points as an incentive for participation. All the procedures adhered to the ethical guidelines of the 1975 Declaration of Helsinki and its subsequent 2013 revision. This study was approved by the Institutional Review Board of Keio University School of Medicine (approval number: 20221120). This study followed the Strengthening the Reporting of Observational Studies in Epidemiology (STROBE) guidelines for cross‐sectional studies (Supplementary Checklist 1). This study also followed the Checklist for Reporting Results of Internet E‐Surveys (CHERRIES) (Supplementary Checklist 2).^27^

## 3 RESULTS

### 3.1 Baseline characteristics

**FIGURE 1:**
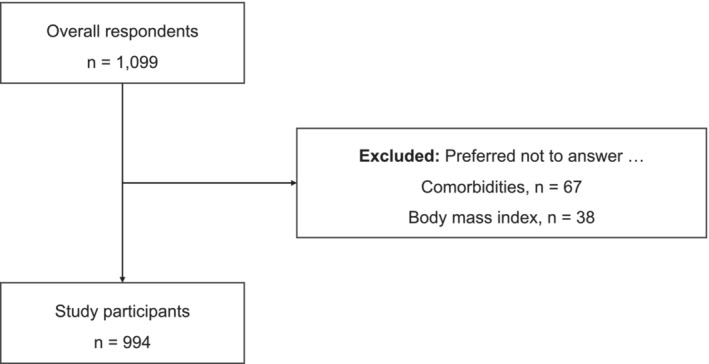
Flow diagram of this study.

Of the 1099 respondents, those who selected ‘prefer not to answer’ for any comorbidity (67 individuals, 6.0%) or for BMI (38 individuals, 3.5%) were excluded, and 994 (90.4%) were included in the analysis (Figure 1). The median age of the participants was 73.2 (SD, 5.3) years, and 516 (51.9%) were male. Regarding socioeconomic indicators, 557 (56.0%) participants had college or higher education. Regarding health‐related factors, 107 (10.8%) participants were current tobacco users, 519 (52.2%) were current alcohol users, 486 (48.9%) had hypertension, 166 (16.7%) had diabetes, 114 (11.5%) had stress urinary incontinence, and 50 (5.0%) had depression. The mean OABSS was 2.7 (SD, 2.2). The mean HUV was 0.90 (SD, 0.11), and the mean EQ VAS score was 75.2 (SD, 17.6) (Table 1). When divided by the presence or absence of OAB, respondents with OAB, compared to those without OAB, were more likely to be male, have a history of smoking, and have comorbidities such as diabetes, stress urinary incontinence, and depression. Those with OAB also had higher OABSS scores and lower HUV than those without OAB (Table S1).

**TABLE 1:** Baseline characteristics of respondents (*n* = 994).

Variables	Category	*n*	%
Age	Mean (SD)	73.2	(5.3)
	Median [IQR]	75	[68–77]
Sex	Male	516	51.9
BMI	Mean (SD)	22.6	(3.4)
	Median [IQR]	22.3	[20.5–24.3]
Educational attainment	College graduate or more	557	56.0
Equivalent household income	Lower half	409	41.2
	Upper half	360	36.2
	Decline to answer	225	22.6
Smoking status	Never	477	48.0
	Past	410	41.3
	Current	107	10.8
Alcohol consumption	Never	246	24.8
	Past	229	23.0
	Current	519	52.2
Presence of comorbidities	Hypertension	486	48.9
	Dyslipidaemia	407	41.0
	Diabetes	166	16.7
	Low back pain	566	56.9
	Ischaemic heart disease	91	9.2
	Stroke	63	6.3
	Chronic kidney disease	36	3.6
	Stress urinary incontinence	114	11.5
	Depression	50	5.0
	Other psychiatric disorders	44	4.4
OABSS	Mean (SD)	2.7	(2.2)
	Median [IQR]	2	[1–4]
Health utility value	Mean (SD)	0.90	(0.11)
	Median [IQR]	0.89	[0.83–1]
EQ‐VAS score	Mean (SD)	75.2	(17.6)
	Median [IQR]	80	[70–88]

Abbreviations: BMI, body mass index; EQ VAS, EuroQol Visual Analogue Scale; IQR, interquartile range; LUTS, lower urinary tract symptom; OABSS, Overactive Bladder Symptom Scale; SD, standard deviation.

### 3.2 Health utility values based on overactive bladder status

Table 2 and Figure S1 present a summary of the HUVs stratified according to the presence and severity of OAB. The mean HUV for participants without OAB was 0.913 (SD, 0.105), whereas that for those with OAB was 0.855 (SD, 0.145). According to the univariate comparison, HUV in those with OAB was lower than those without OAB (β = −0.058, 95% CI ‐0.077 to −0.039, *p* < 0.001). When categorizing OAB severity into mild and moderate‐to‐severe OAB, the mean HUV for participants with mild OAB was 0.874 (SD, 0.131), and for those with moderate‐to‐severe OAB, it was 0.840 (SD, 0.155). Similarly, univariate comparisons showed that HUV in those with mild OAB or moderate‐to‐severe OAB was lower compared to those without OAB (mild OAB: β = −0.039, 95% CI −0.066 to −0.012, *p* = 0.005; moderate‐to‐severe OAB: β = −0.073, 95% CI −0.098 to −0.048, *p* < 0.001).

**TABLE 2:** Prevalence of OAB, utility values, and univariate comparison between non‐OAB and any, mild, moderate‐to‐severe OAB among overall respondents.

OAB severity^a^	*n* (%)	Mean utility value (SD)	Median utility value [IQR]	β	SE	95% CI	*p* value
Non‐OAB	836 (84.1)	0.913 (0.105)	0.894 [0.867–1]				
Any OAB	158 (15.9)	0.855 (0.145)	0.885 [0.784–1]	−0.058	0.010	−0.077 to −0.039	<0.001
Mild OAB	71 (7.1)	0.874 (0.131)	0.895 [0.823–1]	−0.039	0.014	−0.066 to −0.012	0.005
Moderate‐to‐severe OAB	87 (8.8)	0.840 (0.155)	0.844 [0.759–1]	−0.073	0.013	−0.098 to −0.048	<0.001

*Note*: β, SE, 95% CI, and *p* value were estimated using linear regression models.

Abbreviations: CI, confidence interval; IQR, interquartile range; OAB, overactive bladder; SD, standard deviation; SE, standard error.

^a^ Mild OAB is defined as domain 3 (urgency) of overactive bladder symptom score (OABSS) ≥ 2 and total score of 3 ≤ OABSS ≤ 5, and moderate‐to‐severe OAB is defined as domain 3 of OABSS ≥ 2 and total score of 6 ≤ OABSS ≤ 15.

### 3.3 Disutilities of overall respondents

Table 3 presents the results of the multivariable linear regression analyses. After adjusting for age, sex, education, income, smoking status, alcohol use, and presence of comorbidities, the estimated disutility for the presence of OAB was −0.0472 (95% CI, −0.0664 to −0.0279; *p* < 0.001). The estimated disutilities for mild and moderate‐to‐severe OAB were −0.0334 (95% CI, −0.0602 to −0.0066; *p* = 0.014) and −0.0591 (95% CI, −0.0844 to −0.0339; *p* < 0.001), respectively, indicating a linear relationship (*p* for trend <0.001). The sensitivity analysis using the EQ VAS as the outcome variable showed similar results (presence of OAB, β = −5.42, 95% CI −8.47 to −2.37, *p* = 0.001; mild OAB, β = −4.36, 95% CI −8.60 to −0.12, *p* = 0.044; moderate‐to‐severe OAB, β = −6.26, 95% CI −10.26 to −2.27, *p* = 0.002; *p* for trend <0.001).

**TABLE 3:** Results of multivariable linear regression models in the main and sensitivity analyses.

Variables	Category	β^a^	SE	95% CI	*p* value	*p* for trend
Main analysis (outcome measure: EQ‐5D‐5L)						
Presence or absence of OAB						
	Non‐OAB	Reference				Not applicable
	Any OAB	−0.0472	0.0098	−0.0664 to −0.0279	<0.001	
Based on OAB severity						
	Non‐OAB	Reference				<0.001
	Mild OAB	−0.0334	0.0136	−0.0602 to −0.0066	0.014	
	Moderate‐to‐severe OAB	−0.0591	0.0129	−0.0844 to −0.0339	<0.001	

Sensitivity analysis (outcome measure: EQ‐VAS)						
Presence or absence of OAB						
	Non‐OAB	Reference				Not applicable
	Any OAB	−5.42	1.55	−8.47 to −2.37	0.001	
Based on OAB severity						
	Non‐OAB	Reference				<0.001
	Mild OAB	−4.36	2.16	−8.60 to −0.12	0.044	
	Moderate‐to‐severe OAB	−6.26	2.04	−10.26 to −2.27	0.002	

Abbreviations: EQ‐5D‐5L, the 5‐level EuroQoL 5 dimensions version; EQ VAS, EuroQol Visual Analogue Scale; CI, confidence interval; OAB, overactive bladder; SE, standard error.

^a^ Adjusted for age, sex, body mass index, educational attainment, equivalent household income, smoking status, alcohol consumption, and presence of comorbidities (hypertension, dyslipidaemia, diabetes, low back pain, ischaemic heart disease, stroke, chronic kidney disease, stress urinary incontinence, depression, and other psychiatric disorders).

### 3.4 Results of the subgroup analysis

Tables S2 and S3 present the baseline characteristics of the sex and age subgroups, respectively. Table S4 summarizes the HUVs for sex and age subgroups of HUVs. In the sex subgroup, regardless of the presence of OAB, females (*n* = 478) had lower mean HUVs than males (*n* = 516) (e.g. presence of any OAB: male, 0.872 [SD, 0.128]; female, 0.821 [SD, 0.173]). In the age subgroup, regardless of the presence of OAB, the 75–94‐year‐old group (*n* = 502) showed lower HUVs compared with the 65–74‐year‐old group (*n* = 492) (e.g. presence of any OAB: 65–74 years old, 0.889 [SD, 0.130]; 75–94 years old, 0.829 [SD, 0.152]).

**FIGURE 2:**
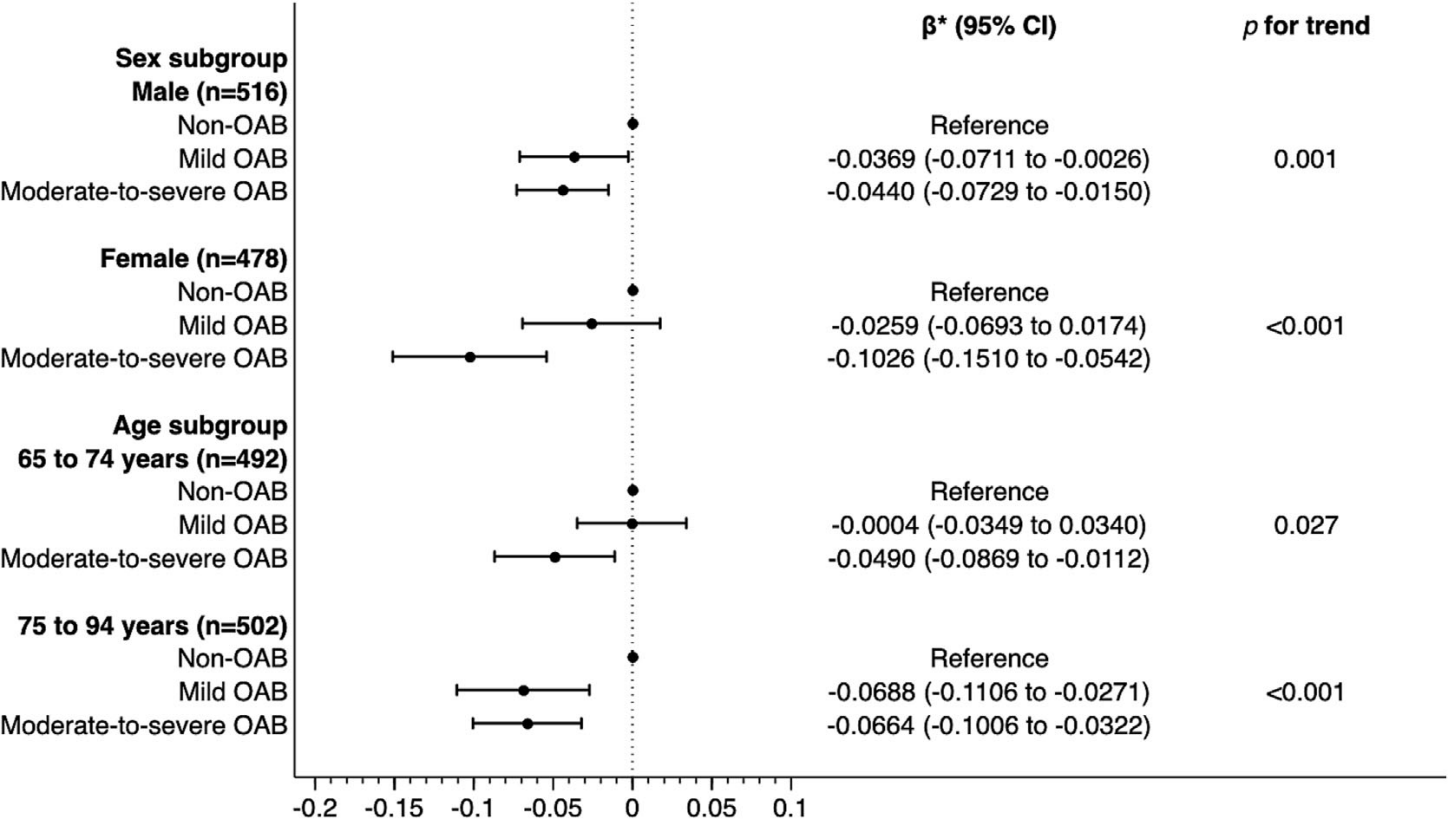
Results of multivariable linear regression models in the subgroup analyses. *Adjusted for age, sex, body mass index, educational attainment, equivalent household income, smoking status, alcohol consumption, and presence of comorbidities (hypertension, dyslipidaemia, diabetes, low back pain, ischaemic heart disease, stroke, chronic kidney disease, stress urinary incontinence, depression, and other psychiatric disorders). Abbreviations: CI, confidence interval; OAB, overactive bladder.

The results of the multivariable linear regression analysis are shown in Figure 2. In the sex subgroup, a substantial disutility was observed in females with moderate‐to‐severe OAB (β = −0.1026; 95% CI, −0.1510 to −0.0542; *p* < 0.001). In the age subgroup, substantial disutilities were observed in the 75–94‐year‐old group with mild OAB (β = −0.0688; 95% CI, −0.1106 to −0.0271; *p* = 0.001) and moderate‐to‐severe OAB (β = −0.0664; 95% CI, −0.1006 to −0.0322; *p* < 0.001).

### 3.5 Results of the exploratory analysis

**FIGURE 3:**
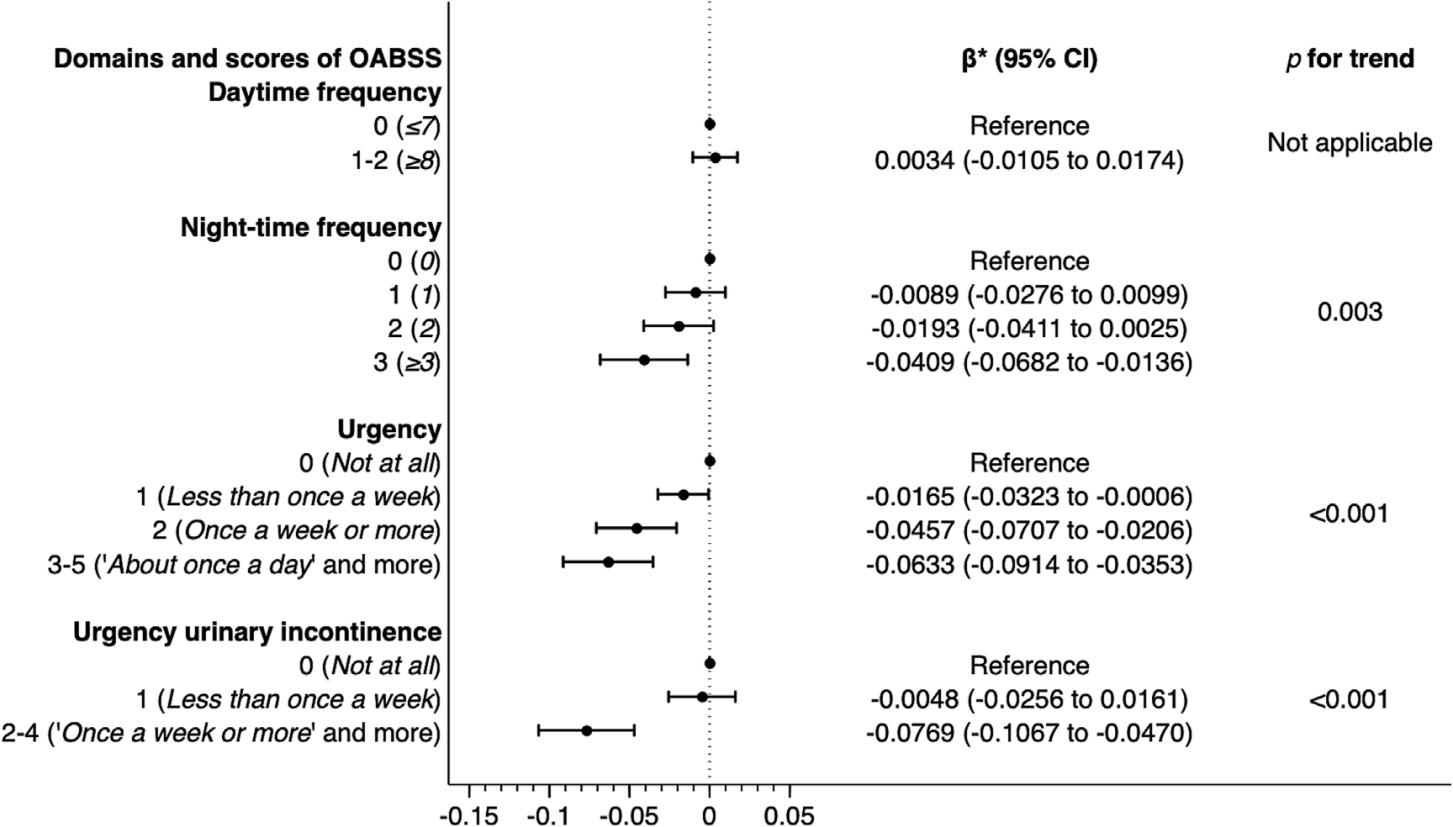
Results of multivariable linear regression models in the exploratory analyses. *Adjusted for age, sex, body mass index, educational attainment, equivalent household income, smoking status, alcohol consumption, and presence of comorbidities (hypertension, dyslipidaemia, diabetes, low back pain, ischaemic heart disease, stroke, chronic kidney disease, stress urinary incontinence, depression, and other psychiatric disorders). Abbreviations: CI, confidence interval; OABSS, overactive bladder symptom score.

Table S5 presents a summary of the HUVs for each domain and the score of the OABSS. The higher OABSS scores indicated the lower mean HUVs in the three domains, excluding urgency urinary incontinence. The results of the multivariable linear regression analysis after rounding scores of <2.5% of the total are shown in Figure 3. After adjusting for covariates, disutilities were observed for each score in the three domains, excluding daytime frequency (e.g. nighttime frequency: 1 point, β = −0.0089, 95% CI −0.0276 to 0.0099, *p* = 0.035; 2 points, β = −0.0193, 95% CI −0.0411 to 0.0025, *p* = 0.082; 3 points, β = −0.0409, 95% CI −0.0682 to −0.0136, *p* = 0.003). A linear relationship between scores and disutilities was observed in all domains, except for daytime frequency (*p* for trend: nighttime frequency, 0.003; urgency, <0.001; and urgency incontinence, <0.001).

## 4 DISCUSSION

This study described the HUVs of Japanese adults aged ≥65 years with or without OAB and estimated the associated disutility of OAB using regression models. The results demonstrated that the mean HUVs of individuals with OAB were lower and the disutility of OAB was greater, depending on the severity of the condition. Subgroup analyses suggested that females and those aged 75 years might experience greater disutility owing to OAB than their counterparts. Furthermore, exploratory analyses indicated the possibility of score‐proportional disutility in all domains of the OABSS, except daytime frequency.

Previous studies have evaluated the associations of OAB with HUVs in several countries. Kay et al. conducted a study estimating EQ‐5D‐3L‐based HUVs from the Incontinence‐Specific Quality of Life Questionnaire in 2505 patients with idiopathic or neurogenic OAB in the United States and Europe.^13^ Similarly, Ruiz et al. used the Overactive Bladder Symptom and Health‐Related Quality of Life Questionnaire in 246 patients with OAB to estimate the effects of OAB symptoms on EQ‐5D‐3L‐based HUVs in Spain.^14^ Additionally, Kim et al. assessed the effects of OAB severity as defined by the OABSS on EQ‐5D‐3L‐based HUVs in 226 867 participants (including 12 303 patients with OAB) from the 2012 Korean Community Health Survey.^15^ These studies shared two common features. First, they estimated the associations of OAB with HUVs using OAB measures that are policy‐relevant in their respective countries. Second, they employed the EQ‐5D‐3L for evaluation. These factors significantly influenced the design of this study in a Japanese setting. Second, regarding the choice of OAB measure, we used the OABSS, similar to Kim et al., as it is explicitly used to define OAB in the Japanese OAB guidelines.^28^ The OABSS was used to define OAB in a randomized controlled trial evaluating the effects of OAB interventions in Japan.^29^ Therefore, decision modelling based on the OABSS is expected when conducting HEEs targeting patients with OAB in Japan. Second, we used the EQ‐5D‐5L, for which a value set developed and validated by Ikeda et al. exists in Japan.^19^ Evidence suggests that the EQ‐5D‐3L may potentially overestimate positive associations and underestimate negative associations compared with the EQ‐5D‐5L in HEEs.^16^ Consequently, the EQ‐5D‐5L is recommended for cost‐effectiveness evaluations in Japan,^30^ and our study adhered to this recommendation. This study, which fully considers Japanese clinical practices and healthcare systems, provides HUVs appropriate for HEEs in the Japanese setting. We believe that our approach offers valuable insights for the design of future studies in any country.

In this study, the disutility values for mild and moderate‐to‐severe OABs as defined by the OABSS were −0.034 and −0.0591, respectively. The disutility that we estimated represents the extent to which OAB affects HUV. Because HUVs and disutilities can be used across different diseases, we can evaluate how much OAB is associated with a decrease in HUV compared to other medical conditions. When referring to the Japanese population norms using the EQ‐5D‐5L, these disutilities are comparable to those of other chronic conditions that affect HRQoL, such as allergic rhinitis (−0.027), atopic dermatitis (−0.031), and other skin diseases (−0.043).^20^ In a report from Korea, which used the same scale (OABSS) and criteria to define OAB as our study, the covariate‐adjusted disutility values for mild OAB, moderate OAB, and severe OAB were −0.029, −0.089, and −0.179, respectively.^15^ The smaller disutility for moderate‐to‐severe OAB in our study compared to Kim et al.'s findings may be explained by differences in the mean age (75 years vs. 45.2 years), outcome measure (EQ‐5D‐5L vs. EQ‐5D‐3L), and ethnicity (Japanese vs. Korean). However, both studies consistently showed that disutility increased with severity, emphasizing the importance of the associations of OAB with HUVs. Furthermore, our study found high disutility (−0.1026) among female participants with moderate‐to‐severe OAB in the subgroup analyses. This finding may be supported by the results of EpiLUTS, an Internet survey conducted among individuals aged >40 years in the United States, which showed that a higher proportion of women (39.4%) than men (28.7%) reported moderate‐to‐severe problems in their patient perception of bladder condition for OAB with bother.^31^ Furthermore, our study's definition of OAB using OABSS might explain the higher disutility observed in women with moderate‐to‐severe OAB. According to evidence from Japan, urgency urinary incontinence, as defined by Q4 of the OABSS, has a significant impact on daily life for women.^32^ In contrast, slow stream and post‐voiding incontinence have been shown to have a greater impact on daily life for men. Patients with moderate‐to‐severe OAB in our study likely have strong symptoms of urgency urinary incontinence, which may have resulted in a larger impact on HUV for women. Additionally, in the subgroup of individuals aged ≥75 years, mild and moderate‐to‐severe OABs were associated with disutility values of similar magnitude (mild OAB −0.0688 vs. moderate‐to‐severe OAB −0.0664). This finding suggests that the presence of OAB, regardless of severity, may have a substantial impact on older adults who are more likely to be frail than younger respondents. For example, evidence has shown that even in the absence of incontinence, the presence of OAB is a significant risk factor for falls.^5^ Furthermore, another hypothetical explanation is that OAB in adults aged ≥75 may be qualitatively different from OAB in those aged 65–74. In fact, among our study participants, those aged ≥75 had higher average scores for nighttime frequency (mean, 65–74 years, 1.096; ≥75 years, 1.402; *p* < 0.001) and urgency urinary incontinence (mean, 65–74 years, 0.185; ≥75 years, 0.301; *p* = 0.003) compared to those aged 65–74. Considering the evidence that bother scores for nighttime frequency and urgency urinary incontinence increase with age,^33^ this difference in the distribution of each domain may be contributing to the larger disutility.

Our exploratory analysis, which examined disutility for each of the four OABSS domains (daytime frequency, nighttime frequency, urgency, and urgency urinary incontinence), is novel and noteworthy. Our results showed a linear association between disutility and scores for nighttime frequency, urgency, and urgency urinary incontinence, suggesting that each of these symptoms may affect HRQoL as defined by EQ‐5D's domains: mobility, self‐care, usual activities, pain/discomfort, and anxiety/depression. Evidence shows that among the OABSS subscales changed after anticholinergic medication for OAB, urgency and urgency urinary incontinence correlated with changes in International Prostate Symptom Score‐QOL and several King's Health Questionnaire subdomains (urgency: impact on life, physical limitations, and emotions; urgency urinary incontinence: role limitations, emotions, and severity measures).^34^ Combining this evidence with our results suggests that OAB domains other than daytime frequency might lead to decreased HUV through limitations in daily activities and worsening of emotional state.

Our study has several strengths. First, we comprehensively provided the descriptive and analytical information necessary for the HEE of OAB, specifically for older adults. The mean and median HUVs at baseline and the disutilities after covariate adjustment for each severity level of OAB are essential information when conducting HEEs. Second, this study estimated disutility by adjusting for a wide range of evidence‐based covariates, from socioeconomic to health‐related indicators. Third, a subgroup analysis was performed to identify vulnerable populations that are essential for HEEs from the perspective of equity considerations but are understudied.^35^ Moreover, our subgroup analysis results provide HUVs that can be referenced in future HEEs, particularly when treatments specifically targeting nocturia or urgency urinary incontinence among the OAB symptoms become available.

However, this study has also some limitations. First, as this study utilized an Internet survey, there were concerns regarding the external validity of the results. Evidence suggests that respondents who use paper rather than online responses are more likely to be female, have lower education levels, be unmarried, be fully retired, have depression, and report poor self‐rated health. Indeed, our study participants had a high proportion of university graduates or higher (56.0%), suggesting that their demographic and socioeconomic background may differ from the general Japanese population.^36^ Furthermore, compared to the general Japanese population, our study participants had a lower proportion of individuals aged 65–74 years, and among those 75 years and older, there was a higher proportion of males. However, the prevalence of OAB (15.9%) and mean HUV (0.89) in this study did not substantially differ from previous epidemiological data from Japan.^20^, ^37^ Thus, these values are also expected to apply to HEEs. Second, the sample size was limited (approximately 1000); consequently, there were few individuals with high scores on the OABSS (e.g. 2 points for daytime frequency and 4–5 points for urgency urinary incontinence). As a result, it was not possible to differentiate between moderate and severe OAB, and some scores were grouped in the exploratory analysis. Although the number of individuals with such high scores may not be large and may not be considered in actual HEEs, revalidation of our study with a larger sample size would be valuable. Third, there may have been residual confounding factors. For example, orthopaedic factors, such as falls and fractures, can be considered in future models.

Our study has clinical implications. Our results suggest that the disease burden of OAB is substantial for older adults, potentially affecting mobility, self‐care, usual activities, pain/discomfort, and anxiety/depression through OAB symptoms. This highlights the importance for clinicians to consider HRQoL, as well as OAB symptoms, as treatment endpoints. Furthermore, our subgroup analysis identified women with moderate‐to‐severe OAB and patients aged ≥75 with mild‐ or moderate‐to‐severe OAB as vulnerable populations with a greater impact on HUV. This emphasizes the importance of promoting OAB awareness and consultation in these groups, as well as the significance of management strategies aimed at preventing the progression of OAB severity.

In conclusion, this study examined HUVs in older Japanese adults with and without OAB. The results also demonstrated that increasing the severity of OAB indicated greater disutility, highlighting the substantial impact of OAB on HRQoL. These findings serve as valuable resources for future HEEs of OAB, possibly guiding decision‐making in healthcare resource allocation for policymakers in Japan. Similar data should be researched and elucidated by other countries. Furthermore, these results emphasize the importance of HUV measurement in future clinical trials aimed at verifying the effects of new therapeutic interventions for OAB.

## AUTHOR CONTRIBUTIONS

**Takashi Yoshioka:** Conceptualization; methodology; software; formal analysis; investigation; resources; data curation; writing—original draft; visualization; supervision; project administration; funding acquisition. **Kenji Omae:** Validation; writing—review and editing. **Satoshi Funada:** Validation; writing—review and editing. **Tetsuji Minami:** Validation; writing—review and editing. **Rei Goto:** Validation; writing—review and editing; funding acquisition.

## CONFLICT OF INTEREST STATEMENT

TY has received the Japan Society for the Promotion of Science (JSPS) KAKENHI grant (grant number: 21K17228) for conducting the survey. RG has received grants for the evaluation of the cost‐effectiveness of medicines and medical devices from the National Institute of Public Health, Japan since 2019, which support the consultation fees for the development of search strategies and will support the article publication fee. KO, SF, and TM have no conflicts of interest to declare. The funders did not participate in the study design, data collection, analysis, interpretation, manuscript preparation, review, approval, or decision to submit the manuscript for publication.

## Supporting information


**Figure S1** Health utility values stratified according to the presence and severity of OAB.
**Table S1** Baseline characteristics according to the OAB status.
**Table S2** Baseline characteristics of the sex subgroups.
**Table S3** Baseline characteristics of the age subgroups.
**Table S4** Prevalence of OAB, utility values, and univariate comparison according to sex or age subgroups.
**Table S5** Proportion and utility values of each score according to the OABSS domains.
**Method S1** The questionnaires and response options in the survey.

## Data Availability

The datasets generated and analysed during the current study are available from the corresponding author upon reasonable request.
