# Supplementary material for: Health utility value of overactive bladder in Japanese older adults
Source: BJUI Compass. 2024 Nov 24;6(1):e471. doi: 10.1002/bco2.471 (PMC11771497; doi:10.1002/bco2.471)
Supplement: Supplementary file 1 — Figure S1 Health utility values stratified according to the presence and severity of OAB. Table S1 Baseline characteristics according to the OAB status. Table S2 Baseline characteristics of the sex subgroups. Table S3 Baseline characteristics of the age subgroups. Table S4 Prevalence of OAB, utility values, and univariate comparison according to sex or age subgroups. Table S5 Proportion and utility values of each score according to the OABSS domains. Method S1 The questionnaires and response options in the survey. [file BCO2-6-e471-s001.docx]

**Supplementary materials**

**Method S1** The questionnaires and response options in the survey

1. What is your age?
2. What is your sex?
   1. Male
   2. Female
   3. Others
3. Please provide your current height and weight.
   1. Height (cm):
   2. Weight (kg):
4. What is your annual household income? Please choose one of the following options:

^*^This includes the total income earned by your entire household in 1 year, including income from work and other sources (such as remittances from children, property income like rent, benefits for older adults).

1. Less than 1 million yen
2. 1 million yen to <2 million yen
3. 2 million yen to <4 million yen
4. 4 million yen to <6 million yen
5. 6 million yen to <10 million yen
6. 10 million yen to <15 million yen
7. 15 million yen to <20 million yen
8. ≥20 million yen
9. Prefer not to answer
10. Do not know
11. Including yourself, how many family members share your household finances?
12. Do you currently have a spouse (husband or wife, including a partner, in a de facto marital relationship)?
    1. No (I am single)
    2. Yes
    3. I had one, but they passed away (widowed).
    4. I had one, but we divorced.
13. Do you currently smoke tobacco habitually?
    1. I smoke almost always.
    2. I smoke occasionally.
    3. I used to smoke in the past, but I do not smoke now.
    4. I do not smoke and have never smoked.
14. Do you currently drink alcohol habitually?
    1. I drink almost always.
    2. I drink occasionally.
    3. I used to drink in the past, but I do not drink now.
    4. I do not drink and have never drunk.
15. Have you experienced any of the following conditions? For each condition, we selected one of the following options:
    1. Never had it
    2. Had (or have) it, but not currently receiving treatment at a hospital
    3. Have it and currently receiving treatment at a hospital
    4. Prefer not to answer

Conditions:

- - 1. Hypertension (high blood pressure)
    2. Diabetes
    3. Hyperlipidemia/hypercholesterolemia
    4. Lower back pain/lumbago
    5. Angina pectoris/myocardial infarction
    6. Cerebral infarction/cerebral hemorrhage
    7. Kidney disease/renal failure
    8. Stress urinary incontinence (involuntary urination when coughing, sneezing, etc.)
    9. Depression
    10. Mental health conditions other than depression (requiring psychiatric or psychosomatic medicine)

OABSS

1. How many times do you typically urinate from waking in the morning until sleeping at night?
   1. ≤7
   2. 8–14
   3. ≥15
2. How many times do you typically wake up to urinate from sleeping at night until waking in the morning?
3. 0
4. 1
5. 2
6. ≥3
7. How often do you have a sudden desire to urinate, which is difficult to defer?
   1. Not at all
   2. Less than once a week
   3. Once a week or more
   4. About once a day
   5. 4 times a day
   6. 5 times a day or more
8. How often do you leak urine because you cannot defer the sudden desire to urinate?
   1. Not at all
   2. Less than once a week
   3. Once a week or more
   4. About once a day
   5. 4 times a day
   6. 5 times a day or more

In addition to the above questions, we used the Japanese versions of the EQ-5D-5L and EQ VAS. For information on the EQ-5D-5L and EQ VAS, please refer to the user guide available on the EuroQol website (https://euroqol. org/information-and-support/documentation/user-guides).

**Table S1** Baseline characteristics according to the OAB status

| **Variables** | **Category** | **OAB, absent (n=836)** | | **OAB, present (n=158)** | | ***p*-value** |
| --- | --- | --- | --- | --- | --- | --- |
|  |  | **n** | **%** | **n** | **%** |  |
| Age | mean (SD) | 73.1 | (5.2) | 73.8 | (5.9) | 0.15 |
|  | median (IQR) | 74 | (68, 76) | 75 | (68, 77) |  |
| Sex | Male | 409 | 48.9 | 107 | 67.7 | <0.001 |
| BMI | mean (SD) | 22.5 | (3.4) | 23. | (3.2) | 0.07 |
|  | median (IQR) | 22.2 | (20.5, 24.2) | 22.9 | (20.8, 24.6) |  |
| Educational attainment | College graduate or more | 476 | 56.9 | 81 | 51.3 | 0.19 |
| Equivalent household income | Lower half | 341 | 40.8 | 68 | 43.0 | 0.18 |
|  | Upper half | 297 | 35.5 | 63 | 39.9 |  |
|  | Decline to answer | 198 | 23.7 | 27 | 17.1 |  |
| Smoking status | Never | 426 | 51.0 | 51 | 32.3 | <0.001 |
|  | Past | 323 | 38.6 | 87 | 55.1 |  |
|  | Current | 87 | 10.4 | 20 | 12.7 |  |
| Alcohol consumption | Never | 215 | 25.7 | 31 | 19.6 | 0.08 |
|  | Past | 183 | 21.9 | 46 | 29.1 |  |
|  | Current | 438 | 52.4 | 81 | 51.3 |  |
| Presence of comorbidities | Hypertension | 399 | 47.7 | 87 | 55.1 | 0.10 |
|  | Dyslipidemia | 341 | 40.8 | 66 | 41.8 | 0.86 |
|  | Diabetes | 127 | 15.2 | 39 | 24.7 | 0.005 |
|  | Low back pain | 465 | 55.6 | 101 | 63.9 | 0.05 |
|  | Ischemic heart disease | 70 | 8.4 | 21 | 13.3 | 0.07 |
|  | Stroke | 48 | 5.7 | 15 | 9.5 | 0.11 |
|  | Chronic kidney disease | 29 | 3.5 | 7 | 4.4 | 0.49 |
|  | Stress urinary incontinence | 77 | 9.2 | 37 | 23.4 | <0.001 |
|  | Depression | 35 | 4.2 | 15 | 9.5 | 0.03 |
|  | Other psychiatric disorders | 35 | 4.2 | 9 | 5.7 | 0.40 |
| OABSS | mean (SD) | 2.0 | (1.4) | 6.3 | (2.1) | <0.001 |
|  | median (IQR) | 2 | (1, 3) | 6 | (5, 8) |  |
| Health utility value | mean (SD) | 0.91 | (0.11) | 0.86 | (0.15) | <0.001 |
|  | median (IQR) | 0.89 | (0.87, 1) | 0.88 | (0.78, 1) |  |

**Note.** BMI, body mass index; IQR, interquartile range; OABSS, overactive bladder symptom scale; SD, standard deviation.

**Table S2** Baseline characteristics of the sex subgroups

| **Variables** | **Category** | **Male (n=516)** | | **Female (n=478)** | |
| --- | --- | --- | --- | --- | --- |
|  |  | **n** | **%** | **n** | **%** |
| Age | Mean (SD) | 73.3 | (5.3) | 73.1 | (5.3) |
|  | Median [IQR] | 75 | [68.5–77] | 74 | [68–76] |
| BMI | Mean (SD) | 23.1 | (2.8) | 21.9 | (3.8) |
|  | Median [IQR] | 22.9 | [21.3–23.7] | 21.6 | [19.7–23.7] |
| Educational attainment | College graduate or more | 322 | 62.4 | 235 | 49.2 |
| Equivalent household income | Lower half | 220 | 42.6 | 189 | 39.5 |
|  | Upper half | 201 | 39.0 | 159 | 33.3 |
|  | Decline to answer | 95 | 18.4 | 130 | 27.2 |
| Smoking status | Never | 97 | 18.8 | 380 | 79.5 |
|  | Past | 341 | 66.1 | 69 | 14.4 |
|  | Current | 78 | 15.1 | 29 | 6.1 |
| Alcohol consumption | Never | 52 | 10.1 | 194 | 40.6 |
|  | Past | 126 | 24.4 | 103 | 21.6 |
|  | Current | 338 | 65.5 | 181 | 37.9 |
| Presence of comorbidities | Hypertension | 288 | 55.8 | 198 | 41.4 |
|  | Dyslipidemia | 196 | 38.0 | 211 | 44.1 |
|  | Diabetes | 130 | 25.2 | 36 | 7.5 |
|  | Low back pain | 316 | 61.2 | 250 | 52.3 |
|  | Ischemic heart disease | 64 | 12.4 | 27 | 5.7 |
|  | Stroke | 48 | 9.3 | 15 | 3.1 |
|  | Chronic kidney disease | 21 | 4.1 | 15 | 3.1 |
|  | Stress urinary incontinence | 22 | 4.3 | 92 | 19.3 |
|  | Depression | 30 | 5.8 | 20 | 4.2 |
|  | Other psychiatric disorders | 21 | 4.1 | 23 | 4.8 |
| OABSS | Mean (SD) | 3.1 | (2.3) | 2.2 | (1.9) |
|  | Median [IQR] | 3 | [1–4] | 2 | [1–3] |
| HUV | Mean (SD) | 0.91 | (0.11) | 0.90 | (0.12) |
|  | Median [IQR] | 0.89 | [0.84–1] | 0.89 | [0.83–1] |

**Note.** BMI, body mass index; HUV, health utility value; IQR, interquartile range; OABSS, Overactive Bladder Symptom Scale; SD, standard deviation.

**Table S3** Baseline characteristics of the age subgroups

| **Variables** | **Category** | **65 to 74 (n=492)** | | **75 to 94 (n=502)** | |
| --- | --- | --- | --- | --- | --- |
|  |  | **n** | **%** | **n** | **%** |
| Age | Mean (SD) | 68.7 | (2.9) | 77.7 | (2.9) |
|  | Median [IQR] | 68 | [66–71] | 77 | [75–79] |
| Sex | Male | 250 | 50.8 | 266 | 53.0 |
| BMI | Mean (SD) | 22.5 | (3.2) | 22.6 | (3.5) |
|  | Median [IQR] | 22.5 | [20.3–24.3] | 22.2 | [20.8–24.3] |
| Educational attainment | College graduate or more | 309 | 62.8 | 248 | 49.4 |
| Equivalent household income | Lower half | 183 | 37.2 | 226 | 45.0 |
|  | Upper half | 184 | 37.4 | 176 | 35.1 |
|  | Decline to answer | 125 | 25.4 | 100 | 19.9 |
| Smoking status | Never | 229 | 46.5 | 248 | 49.4 |
|  | Past | 197 | 40.0 | 213 | 42.4 |
|  | Current | 66 | 13.4 | 41 | 8.2 |
| Alcohol consumption | Never | 115 | 23.4 | 131 | 26.1 |
|  | Past | 116 | 23.6 | 113 | 22.5 |
|  | Current | 261 | 53.1 | 258 | 51.4 |
| Presence of comorbidities | Hypertension | 214 | 43.5 | 272 | 54.2 |
|  | Dyslipidemia | 203 | 41.3 | 204 | 40.6 |
|  | Diabetes | 83 | 16.9 | 83 | 16.5 |
|  | Low back pain | 273 | 55.5 | 293 | 58.4 |
|  | Ischemic heart disease | 39 | 7.9 | 52 | 10.4 |
|  | Stroke | 28 | 5.7 | 35 | 7.0 |
|  | Chronic kidney disease | 13 | 2.6 | 23 | 4.6 |
|  | Stress urinary incontinence | 52 | 10.6 | 62 | 12.4 |
|  | Depression | 30 | 6.1 | 20 | 4.0 |
|  | Other psychiatric disorders | 25 | 5.1 | 19 | 3.8 |
| OABSS | Mean (SD) | 2.4 | (2.0) | 2.9 | (2.3) |
|  | Median [IQR] | 2 | [1–3] | 2 | [1–4] |
| HUV | Mean (SD) | 0.92 | (0.10) | 0.89 | (0.12) |
|  | Median [IQR] | 0.89 | [0.87–1] | 0.89 | [0.83–1] |

**Note.** BMI, body mass index; HUV, health utility value; IQR, interquartile range; OABSS, Overactive Bladder Symptom Scale; SD, standard deviation.

**Table S4** Prevalence of OAB, utility values, and univariate comparison according to sex or age subgroups

| **Population** | **OAB severity^*^** | **n (%)** | **Mean HUV (SD)** | **Median HUV [IQR]** | **β** | **SE** | **95% CI** | ***p*-value** |
| --- | --- | --- | --- | --- | --- | --- | --- | --- |
| **Sex subgroups** |  |  |  |  |  |  |  |  |
| **Male (n=516)** | **Non-OAB** | **409 (79.3)** | **0.917 (0.104)** | **0.895 [0.875–1]** | **Reference** | | | |
|  | **Any OAB** | **107 (20.7)** | **0.872 (0.128)** | **0.895 [0.823–1]** | **-0.045** | **0.012** | **-0.069 to -0.022** | **<0.001** |
|  | Mild OAB | 43 (8.3) | 0.882 (0.119) | 0.895 [0.823–1] | -0.035 | 0.017 | -0.070 to -0.001 | 0.043 |
|  | Moderate-to-severe OAB | 64 (12.4) | 0.865 (0.134) | 0.895 [0.811–1] | -0.052 | 0.015 | -0.081 to -0.023 | <0.001 |
| **Female (n=478)** | **Non-OAB** | **427 (89.3)** | **0.909 (0.107)** | **0.894 [0.867–1]** | **Reference** | | | |
|  | **Any OAB** | **51 (10.7)** | **0.821 (0.173)** | **0.831 [0.759–0.895]** | **-0.089** | **0.017** | **-0.122 to -0.055** | **<0.001** |
|  | Mild OAB | 28 (5.9) | 0.862 (0.150) | 0.895 [0.824–1] | -0.047 | 0.022 | -0.091 to -0.003 | 0.035 |
|  | Moderate-to-severe OAB | 23 (4.8) | 0.770 (0.189) | 0.823 [0.670–0.895] | -0.139 | 0.025 | -0.187 to -0.091 | <0.001 |
| **Age subgroups** |  |  |  |  |  |  |  |  |
| **65–74 years (n=492)** | **Non-OAB** | **423 (86.0)** | **0.920 (0.100)** | **0.895 [0.875–1]** |  |  |  |  |
|  | **Any OAB** | **69 (14.0)** | **0.889 (0.130)** | **0.895 [0.823–1]** | **-0.031** | **0.014** | **-0.057 to -0.004** | **0.024** |
|  | Mild OAB | 37 (7.5) | 0.916 (0.091) | 0.895 [0.829–1] | -0.004 | 0.018 | -0.039 to 0.031 | 0.833 |
|  | Moderate-to-severe OAB | 32 (6.5) | 0.858 (0.160) | 0.895 [0.766–1] | 0.062 | 0.019 | -0.099 to -0.024 | 0.001 |
| **75–94 years (n=502)** | **Non-OAB** | **413 (82.3)** | **0.907 (0.111)** | **0.894 [0.867–1]** |  |  |  |  |
|  | **Any OAB** | **89 (17.7)** | **0.829 (0.152)** | **0.831 [0.776–0.895]** | **-0.078** | **0.014** | **-0.105 to -0.050** | **<0.001** |
|  | Mild OAB | 34 (6.8) | 0.829 (0.153) | 0.849 [0.780–0.895] | -0.078 | 0.021 | -0.120 to 0.036 | <0.001 |
|  | Moderate-to-severe OAB | 55 (11.0) | 0.830 (0.152) | 0.831 [0.759–1] | -0.077 | 0.017 | -0.111 to 0.044 | <0.001 |

**Note.** CI, confidence interval; HUV, health utility value; IQR, interquartile range; OAB, overactive bladder; SD, standard deviation; SE, standard error. β, SE, 95% CI, and *p*-value were estimated using linear regression models.

^*^Mild OAB is defined as domain 3 (urgency) of overactive bladder symptom score (OABSS)≥2 and total score of 3≤OABSS≤5, and moderate-to-severe OAB is defined as domain 3 of OABSS≥2 and total score of 6≤OABSS≤15.

**Table S5** Proportion and utility values of each score according to the OABSS domains

| **Domain** | **Score** | **n (%)** | **Mean HUV (SD)** | **Median HUV [IQR]** |
| --- | --- | --- | --- | --- |
| **Daytime frequency** | 0 | 549 (55.2) | 0.905 (0.113) | 0.895 [0.831–1] |
|  | 1 | 430 (43.3) | 0.905 (0.114) | 0.895 [0.844–1] |
|  | 2 | 15 (1.5) | 0.854 (0.158) | 0.895 [0.759–1] |
| **Nighttime frequency** | 0 | 195 (19.6) | 0.924 (0.096) | 1 [0.895–1] |
|  | 1 | 463 (46.6) | 0.909 (0.106) | 0.895 [0.867–1] |
|  | 2 | 228 (22.9) | 0.894 (0.116) | 0.895 [0.831–1] |
|  | 3 | 108 (10.9) | 0.869 (0.160) | 0.895 [0.823–1] |
| **Urgency** | 0 | 557 (56.0) | 0.920 (0.096) | 0.895 [0.875–1] |
|  | 1 | 279 (28.1) | 0.899 (0.121) | 0.895 [0.823–1] |
|  | 2 | 88 (8.9) | 0.866 (0.150) | 0.895 [0.823–1] |
|  | 3 | 42 (4.2) | 0.843 (0.141) | 0.873 [0.772–1] |
|  | 4 | 22 (2.2) | 0.842 (0.148) | 0.856 [0.759–1] |
|  | 5 | 6 (0.6) | 0.825 (0.117) | 0.831 [0.708–0.895] |
| **Urgency incontinence** | 0 | 818 (82.3) | 0.910 (0.108) | 0.895 [0.867–1] |
|  | 1 | 132 (13.3) | 0.896 (0.120) | 0.895 [0.831–1] |
|  | 2 | 27 (2.7) | 0.804 (0.175) | 0.831 [0.708–0.895] |
|  | 3 | 12 (1.2) | 0.842 (0.139) | 0.881 [0.741–0.947] |
|  | 4 | 5 (0.5) | 0.753 (0.185) | 0.759 [0.641–0.844] |
|  | 5 | 0 (0) | 0 (0) | 0 [0–0] |

**Note.** HUV, health utility value; IQR, interquartile range; OABSS, Overactive Bladder Symptom Score; SD, standard deviation.

**Figure S1** Health utility values stratified according to the presence and severity of OAB


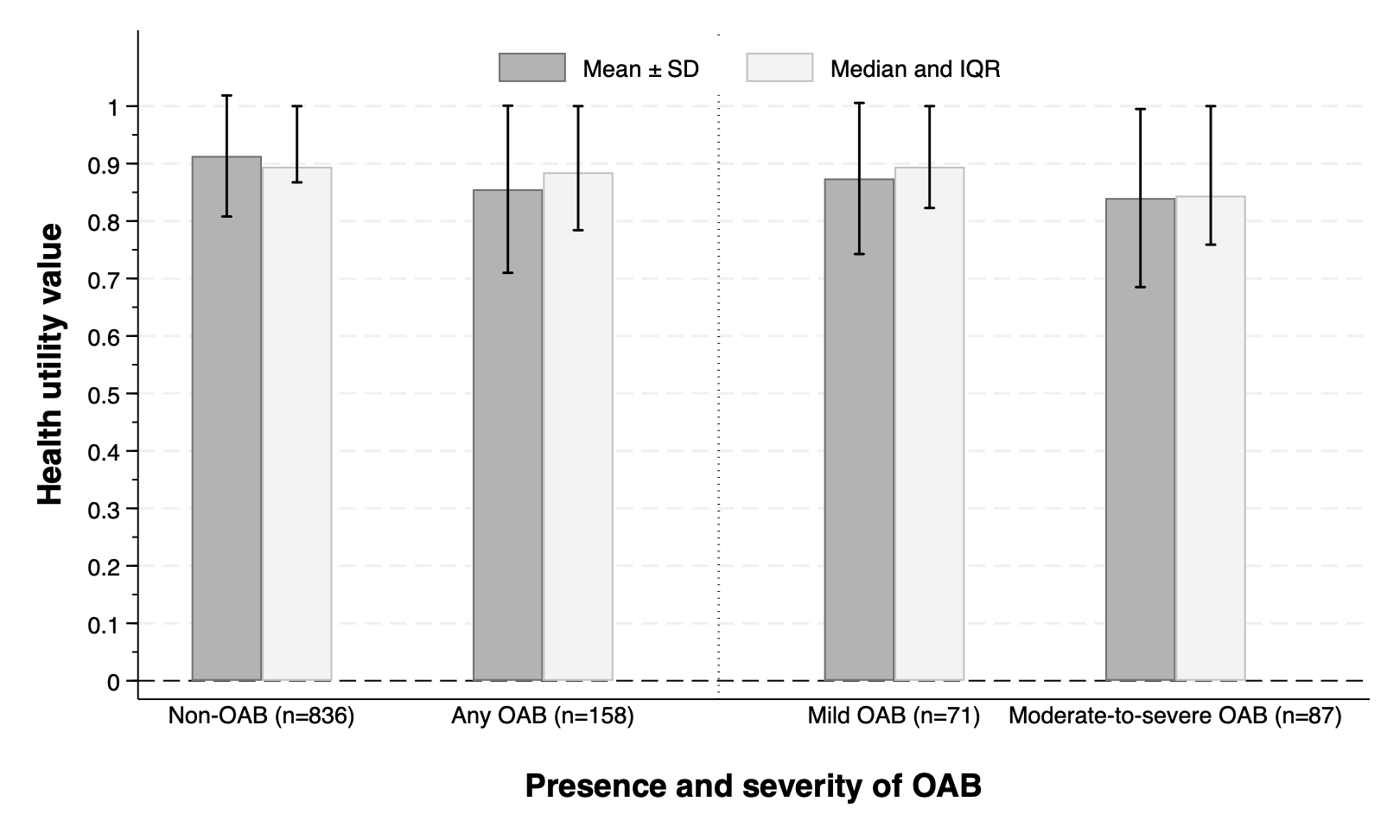


**Note.** OAB, overactive bladder; IQR, interquartile range; SD, standard deviation. The error bars in the mean bar chart represent ±SD, while the error bars in the median chart represent IQR.

**Supplementary Checklist 1**

STROBE Statement—checklist of items that should be included in reports of observational studies

|  | Item No. | Recommendation | Page  No. | Relevant text from manuscript |
| --- | --- | --- | --- | --- |
| **Title and abstract** | 1 | (*a*) Indicate the study’s design with a commonly used term in the title or the abstract | 1 |  |
|  |  | (*b*) Provide in the abstract an informative and balanced summary of what was done and what was found | 3 |  |
| Introduction | | | |  |
| Background/rationale | 2 | Explain the scientific background and rationale for the investigation being reported | 5-6 |  |
| Objectives | 3 | State specific objectives, including any prespecified hypotheses | 6 |  |
| Methods | | | |  |
| Study design | 4 | Present key elements of study design early in the paper | 6 |  |
| Setting | 5 | Describe the setting, locations, and relevant dates, including periods of recruitment, exposure, follow-up, and data collection | 7-8 |  |
| Participants | 6 | (*a*) *Cohort study*—Give the eligibility criteria, and the sources and methods of selection of participants. Describe methods of follow-up  *Case-control study*—Give the eligibility criteria, and the sources and methods of case ascertainment and control selection. Give the rationale for the choice of cases and controls  *Cross-sectional study*—Give the eligibility criteria, and the sources and methods of selection of participants | 8 |  |
|  |  | (*b*) *Cohort study*—For matched studies, give matching criteria and number of exposed and unexposed  *Case-control study*—For matched studies, give matching criteria and the number of controls per case | N/A |  |
| Variables | 7 | Clearly define all outcomes, exposures, predictors, potential confounders, and effect modifiers. Give diagnostic criteria, if applicable | 8-10 |  |
| Data sources/ measurement | 8* | For each variable of interest, give sources of data and details of methods of assessment (measurement). Describe comparability of assessment methods if there is more than one group | 8-10 |  |
| Bias | 9 | Describe any efforts to address potential sources of bias | 7, Method S1 |  |
| Study size | 10 | Explain how the study size was arrived at | N/A |  |

Continued on next page

| Quantitative variables | 11 | Explain how quantitative variables were handled in the analyses. If applicable, describe which groupings were chosen and why | 7-9 |  |
| --- | --- | --- | --- | --- |
| Statistical methods | 12 | (*a*) Describe all statistical methods, including those used to control for confounding | 10-11 |  |
|  |  | (*b*) Describe any methods used to examine subgroups and interactions | N/A |  |
|  |  | (*c*) Explain how missing data were addressed | 7 |  |
|  |  | (*d*) *Cohort study*—If applicable, explain how loss to follow-up was addressed  *Case-control study*—If applicable, explain how matching of cases and controls was addressed  *Cross-sectional study*—If applicable, describe analytical methods taking account of sampling strategy | 7 |  |
|  |  | (*e*) Describe any sensitivity analyses | 10-11 |  |
| Results | | | | |
| Participants | 13* | (a) Report numbers of individuals at each stage of study—eg numbers potentially eligible, examined for eligibility, confirmed eligible, included in the study, completing follow-up, and analysed | 11-12, Figure 1 |  |
|  |  | (b) Give reasons for non-participation at each stage | 11, Figure 1 |  |
|  |  | (c) Consider use of a flow diagram | Figure 1 |  |
| Descriptive data | 14* | (a) Give characteristics of study participants (eg demographic, clinical, social) and information on exposures and potential confounders | 11-12, Table 1 |  |
|  |  | (b) Indicate number of participants with missing data for each variable of interest | N/A |  |
|  |  | (c) *Cohort study*—Summarise follow-up time (eg, average and total amount) | N/A |  |
| Outcome data | 15* | *Cohort study*—Report numbers of outcome events or summary measures over time | N/A |  |
|  |  | *Case-control study—*Report numbers in each exposure category, or summary measures of exposure | N/A |  |
|  |  | *Cross-sectional study—*Report numbers of outcome events or summary measures | 12, Table 2 |  |
| Main results | 16 | (*a*) Give unadjusted estimates and, if applicable, confounder-adjusted estimates and their precision (eg, 95% confidence interval). Make clear which confounders were adjusted for and why they were included | 12, Table S1 |  |
|  |  | (*b*) Report category boundaries when continuous variables were categorized | Table 1, |  |
|  |  | (*c*) If relevant, consider translating estimates of relative risk into absolute risk for a meaningful time period | N/A |  |

Continued on next page

| Other analyses | 17 | Report other analyses done—eg analyses of subgroups and interactions, and sensitivity analyses | 13-14, Table S2-S5, Figure 2-3 |  |
| --- | --- | --- | --- | --- |
| Discussion | | | | |
| Key results | 18 | Summarise key results with reference to study objectives | 14 |  |
| Limitations | 19 | Discuss limitations of the study, taking into account sources of potential bias or imprecision. Discuss both direction and magnitude of any potential bias | 17-18 |  |
| Interpretation | 20 | Give a cautious overall interpretation of results considering objectives, limitations, multiplicity of analyses, results from similar studies, and other relevant evidence | 15-18 |  |
| Generalisability | 21 | Discuss the generalisability (external validity) of the study results | 18 |  |
| Other information | |  | | |
| Funding | 22 | Give the source of funding and the role of the funders for the present study and, if applicable, for the original study on which the present article is based | 19 |  |

*Give information separately for cases and controls in case-control studies and, if applicable, for exposed and unexposed groups in cohort and cross-sectional studies.

**Note:** An Explanation and Elaboration article discusses each checklist item and gives methodological background and published examples of transparent reporting. The STROBE checklist is best used in conjunction with this article (freely available on the Web sites of PLoS Medicine at http://www.plosmedicine.org/, Annals of Internal Medicine at http://www.annals.org/, and Epidemiology at http://www.epidem.com/). Information on the STROBE Initiative is available at www.strobe-statement.org

**Supplementary Checklist 2**

**Checklist for Reporting Results of Internet E-Surveys (CHERRIES)**

| ***Checklist Item*** | ***Explanation*** | ***Page Number*** |
| --- | --- | --- |
| Describe survey design | Describe target population, sample frame. Is the sample a convenience sample? (In “open” surveys this is most likely.) | 7 |
| IRB approval | Mention whether the study has been approved by an IRB. | 10 |
| Informed consent | Describe the informed consent process. Where were the participants told the length of time of the survey, which data were stored and where and for how long, who the investigator was, and the purpose of the study? | 10 |
| Data protection | If any personal information was collected or stored, describe what mechanisms were used to protect unauthorized access. | 7 |
| Development and testing | State how the survey was developed, including whether the usability and technical functionality of the electronic questionnaire had been tested before fielding the questionnaire. | 7 |
| Open survey versus closed survey | An “open survey” is a survey open for each visitor of a site, while a closed survey is only open to a sample which the investigator knows (password-protected survey). | 7 |
| Contact mode | Indicate whether or not the initial contact with the potential participants was made on the Internet. (Investigators may also send out questionnaires by mail and allow for Web-based data entry.) | 7 |
| Advertising the survey | How/where was the survey announced or advertised? Some examples are offline media (newspapers), or online (mailing lists – If yes, which ones?) or banner ads (Where were these banner ads posted and what did they look like?). It is important to know the wording of the announcement as it will heavily influence who chooses to participate. Ideally the survey announcement should be published as an appendix. | N/A |
| Web/E-mail | State the type of e-survey (eg, one posted on a Web site, or one sent out through e-mail). If it is an e-mail survey, were the responses entered manually into a database, or was there an automatic method for capturing responses? |  |
| Context | Describe the Web site (for mailing list/newsgroup) in which the survey was posted. What is the Web site about, who is visiting it, what are visitors normally looking for? Discuss to what degree the content of the Web site could pre-select the sample or influence the results. For example, a survey about vaccination on a anti-immunization Web site will have different results from a Web survey conducted on a government Web site | N/A |
| Mandatory/voluntary | Was it a mandatory survey to be filled in by every visitor who wanted to enter the Web site, or was it a voluntary survey? |  |
| Incentives | Were any incentives offered (eg, monetary, prizes, or non-monetary incentives such as an offer to provide the survey results)? | 10 |
| Time/Date | In what timeframe were the data collected? | 7 |
| Randomization of items or questionnaires | To prevent biases items can be randomized or alternated. | 8 |
| Adaptive questioning | Use adaptive questioning (certain items, or only conditionally displayed based on responses to other items) to reduce number and complexity of the questions. | 7-8 |
| Number of Items | What was the number of questionnaire items per page? The number of items is an important factor for the completion rate. | 8 |
| Number of screens (pages) | Over how many pages was the questionnaire distributed? The number of items is an important factor for the completion rate. | 8 |
| Completeness check | It is technically possible to do consistency or completeness checks before the questionnaire is submitted. Was this done, and if “yes”, how (usually JAVAScript)? An alternative is to check for completeness after the questionnaire has been submitted (and highlight mandatory items). If this has been done, it should be reported. All items should provide a non-response option such as “not applicable” or “rather not say”, and selection of one response option should be enforced. | 8 |
| Review step | State whether respondents were able to review and change their answers (eg, through a Back button or a Review step which displays a summary of the responses and asks the respondents if they are correct). | 8 |
| Unique site visitor | If you provide view rates or participation rates, you need to define how you determined a unique visitor. There are different techniques available, based on IP addresses or cookies or both. | 8 |
| View rate (Ratio of unique survey visitors/unique site visitors) | Requires counting unique visitors to the first page of the survey, divided by the number of unique site visitors (not page views!). It is not unusual to have view rates of less than 0.1 % if the survey is voluntary. | 8 |
| Participation rate (Ratio of unique visitors who agreed to participate/unique first survey page visitors) | Count the unique number of people who filled in the first survey page (or agreed to participate, for example by checking a checkbox), divided by visitors who visit the first page of the survey (or the informed consents page, if present). This can also be called “recruitment” rate. | 8 |
| Completion rate (Ratio of users who finished the survey/users who agreed to participate) | The number of people submitting the last questionnaire page, divided by the number of people who agreed to participate (or submitted the first survey page). This is only relevant if there is a separate “informed consent” page or if the survey goes over several pages. This is a measure for attrition. Note that “completion” can involve leaving questionnaire items blank. This is not a measure for how completely questionnaires were filled in. (If you need a measure for this, use the word “completeness rate”.) | 8 |
| Cookies used | Indicate whether cookies were used to assign a unique user identifier to each client computer. If so, mention the page on which the cookie was set and read, and how long the cookie was valid. Were duplicate entries avoided by preventing users access to the survey twice; or were duplicate database entries having the same user ID eliminated before analysis? In the latter case, which entries were kept for analysis (eg, the first entry or the most recent)? | 8 |
| IP check | Indicate whether the IP address of the client computer was used to identify potential duplicate entries from the same user. If so, mention the period of time for which no two entries from the same IP address were allowed (eg, 24 hours). Were duplicate entries avoided by preventing users with the same IP address access to the survey twice; or were duplicate database entries having the same IP address within a given period of time eliminated before analysis? If the latter, which entries were kept for analysis (eg, the first entry or the most recent)? | 8 |
| Log file analysis | Indicate whether other techniques to analyze the log file for identification of multiple entries were used. If so, please describe. | 8 |
| Registration | In “closed” (non-open) surveys, users need to login first and it is easier to prevent duplicate entries from the same user. Describe how this was done. For example, was the survey never displayed a second time once the user had filled it in, or was the username stored together with the survey results and later eliminated? If the latter, which entries were kept for analysis (eg, the first entry or the most recent)? | 8 |
| Handling of incomplete questionnaires | Were only completed questionnaires analyzed? Were questionnaires which terminated early (where, for example, users did not go through all questionnaire pages) also analyzed? | 8 |
| Questionnaires submitted with an atypical timestamp | Some investigators may measure the time people needed to fill in a questionnaire and exclude questionnaires that were submitted too soon. Specify the timeframe that was used as a cut-off point, and describe how this point was determined. | 8 |
| Statistical correction | Indicate whether any methods such as weighting of items or propensity scores have been used to adjust for the non-representative sample; if so, please describe the methods. | 8 |

This checklist has been modified from Eysenbach G. Improving the quality of Web surveys: the Checklist for Reporting Results of Internet E-Surveys (CHERRIES). J Med Internet Res. 2004 Sep 29;6(3):e34 [erratum in J Med Internet Res. 2012; 14(1): e8.]. Article available at [https://www.jmir.org/2004/3/e34](https://www.jmir.org/2004/3/e34/)/; erratum available <https://www.jmir.org/2012/1/e8/>. Copyright ©Gunther Eysenbach. Originally published in the [Journal of Medical Internet](http://www.jmir.org) Research, 29.9.2004 and 04.01.2012.

This is an open-access article distributed under the terms of the Creative Commons Attribution License (<https://creativecommons.org/licenses/by/2.0/>), which permits unrestricted use, distribution, and reproduction in any medium, provided the original work, first published in the Journal of Medical Internet Research, is properly cited.
